# Novel Compound Heterozygous Pathogenic Variants in *SUOX* Cause Isolated Sulfite Oxidase Deficiency in a Chinese Han Family

**DOI:** 10.3389/fgene.2021.607085

**Published:** 2021-05-07

**Authors:** Jiangang Zhao, Yao An, Haoxiang Jiang, Haibin Wu, Fengyu Che, Ying Yang

**Affiliations:** ^1^Department of Neonatology, Xi’an Children’s Hospital, Xi’an, China; ^2^Department of Radiology, Xi’an Children’s Hospital, Xi’an, China; ^3^Shaanxi Institute for Pediatric Diseases, Xi’an Children’s Hospital, Xi’an, China

**Keywords:** isolated sulfite oxidase deficiency, *SUOX*, molecular diagnosis, infant, neurometabolic disease, genetic counseling

## Abstract

**Aim:**

To explore the clinical imaging, laboratory and genetic characteristics of a newborn boy with isolated sulfite oxidase deficiency (ISOD) in a Chinese mainland cohort.

**Methods:**

Homocysteine and uric acid in plasma and cysteine and total homocysteine in the blood spot were assessed in a Chinese newborn patient with progressive encephalopathy, tonic seizures, abnormal muscle tone, and feeding difficulties. Whole exome sequencing and Sanger sequencing facilitated an accurate diagnosis. The pathogenicity predictions and conservation analysis of the identified mutations were conducted by bioinformatics tools.

**Results:**

Low total homocysteine was detected in the blood spot, while homocysteine and uric acid levels were normal in the plasma. *S*-sulfocysteine was abnormally elevated in urine. A follow-up examination revealed several progressive neuropathological findings. Also, intermittent convulsions and axial dystonia were observed. However, the coordination of sucking and swallowing was slightly improved. A novel paternal nonsense variant c.475G > T (p.Glu159^∗^) and a novel maternal missense variant c.1201A > G (p.Lys401Glu) in *SUOX* were identified in this case by co-segregation verification.

**Conclusion:**

This is the second report of early-onset ISOD case in a non-consanguineous Chinese mainland family. Combined with the clinical characteristics and biochemical indexes, we speculated that these two novel pathogenic variants of the *SUOX* gene underlie the cause of the disease in this patient. Next-generation sequencing (NGS) and Sanger sequencing provided reliable basis for clinical and prenatal diagnoses of this family, it also enriched the mutation spectrum of the *SUOX* gene.

## Introduction

Isolated sulfite oxidase deficiency (ISOD, OMIM: 272300) is an autosomal recessive inherited neurometabolic disease caused by deficient activity of sulfite oxidase. It is characterized by some severe neurological symptoms, including seizures, often non-effective to anticonvulsant medications, and rapidly progressive encephalopathy resulting in a similar condition of neonatal hypoxic ischemia. The majority of the patients developed microcephaly, feeding difficulties, and dislocated ocular lenses. Tissue accumulation and high urinary excretion of sulfite, thiosulfate, and *S*-sulfocysteine were the main biochemical features of the disease (Tan et al., 2005). The time of onset is neonatal or early infantile period. The incidence of ISOD has not been reported epidemiologically. To date, < 50 cases have been reported worldwide (van der Klei-van Moorsel et al., 1991; Rupar et al., 1996; Garrett et al., 1998; Johnson et al., 2002a; Lee et al., 2002; Seidahmed et al., 2005; Claerhout et al., 2018; Chen et al., 2014; Rocha et al., 2014; Zaki et al., 2016; Brumaru et al., 2017; Lee et al., 2017; Mhanni et al., 2020; Sharawat et al., 2020; Du et al., 2021). Recently, four early-onset ISOD patients have been reported in Hong Kong and Taiwan, China (Chan et al., 2002; Lee et al., 2002, 2017; Chen et al., 2014), one early-onset patient in Chinese mainland (Du et al., 2021), and one late-onset ISOD pedigree including three patients have been reported in Chinese mainland (Tian et al., 2019).

Oxidation of sulfite is catalyzed by sulfite oxidase (SO) to sulfate, which constitutes the terminal reaction in the oxidative degradation of sulfur-containing amino acids, methionine, and cysteine. SO is a molybdo hemoprotein comprising of 545 amino acids. The gene encoding SO (*SUOX*, OMIM 606887) maps to chromosome 12q13.2-12q13.3, and the coding sequence contains three exons and two introns (Johnson et al., 2002a). To date, only 29 *SUOX* variants were reported in HGMD database, including missense, nonsense, and deletion, or insertion mutations, which have been identified in unrelated individuals with ISOD worldwide. However, only 5/29 mutations were reported in Taiwan patients (Chen et al., 2014). In this study, we presented the clinical, imaging, and biochemical characteristics of an 18-day-old newborn boy with SO deficiency in the mainland Chinese cohort and two previously unreported pathogenic variants in the *SUOX* gene. The patient was diagnosed based on the clinical features and genetic analysis.

## Materials and Methods

### Clinical Features and Biochemical Findings of the Patient

The proband was a male child born to non-consanguineous chinese parents with a full-term gestation and a vaginal delivery. He had a normal weight (3,020 g) and head circumference (34 cm) at birth. The family history was unremarkable. All members of his family participated in this study after providing written informed consent. The Ethics Committee of the Xi’an Children’s Hospital reviewed and approved our study protocol that was in compliance with the Helsinki declaration.

**FIGURE 1:**
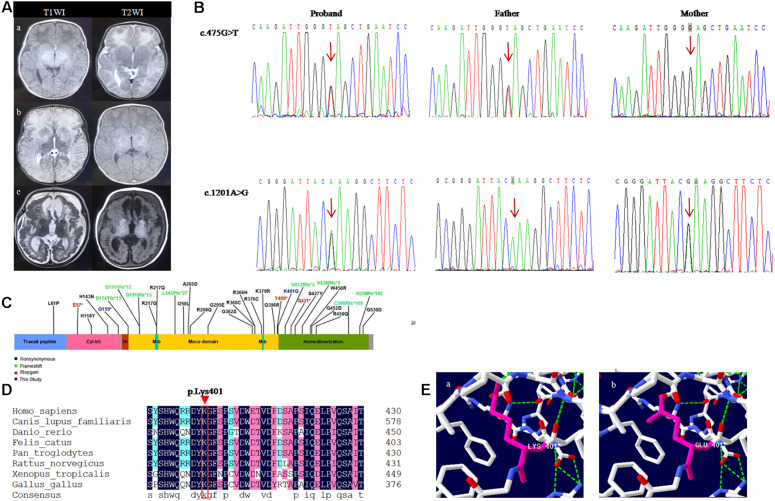
Shows the neuroradiological features and genetic results of the patient. **(A)** Brain MRI of the follow-up of a child with sulfite deficiency enzyme. **(Aa)** MRI findings were normal at the age of 18 days; **(Ab)** 1 month and 3 days after birth, MRI showed high signal on T2WI and low signal onT1WI in bilateral cerebral hemispheres, basal ganglia, and thalamus, DWI showed high signal, and ADC showed low signal; **(Ac)** Follow-up to 5 months, MRI showed polycystic encephalomalacia and atrophy with bilateral subdural effusion. **(B)** Sanger sequencing analysis of *SUOX* gene exon 6 in genomic DNA from the family. **(C)** Linear map of the mutations in *SUOX*. **(D)** Conservation of the p.Lys401Glu variant found in this study. **(E)** Amino acid and conformation changes of the p.Lys401Glu polypeptide wild-type **(Ea)** and mutant type **(Eb)**.

The proband had projectile vomitting at the age of 16 days, accompanied by irritable crying, fever, and diarrhea. After 2 days, he was admitted for further treatment, wherein cardiovascular, abdominal, genitourinary, electrolytes, hepatic, and renal functions were found to be normal except abundant leukocytes detected in the urine routine. The results of blood tandem mass spectrometry analysis were normal. Urine organic acidemia screening showed slightly elevated 3-hydroxypropionic acid, 4-hydroxyphenylacetic acid, and 4-hydroxyphenyl-lactic acid. The day after the admission, he presented enophthalmos in the crying or quiet state. Brain magnetic resonance imaging (MRI) did not show any significant abnormality (Figure 1Aa). The visual evoked potential showed decreased binocular amplitude and prolonged latency. The physicians suspected diarrhea and urinary tract infections, which could be treated before discharge from the hospital.

At the age of 33 days, he was readmitted for fever and diarrhea, which rapidly progressed to encephalopathy, including tonic seizures, unconsciousness, dyspnea, and lethargy. Physical examination did not reveal dysmorphia. The birth weight increased only 230 g in 1 month. Moreover, the patient was irritable, hypertonic, and his coordination of sucking and swallowing was severely impaired (Table 1). Blood and cerebrospinal fluid cultures yielded negative results. Also, the serum ammonia and lactic acid level were significantly elevated. Electro encephalogram (EEG) showed moderately abnormal neonatal data: multifocal sharp waves and frequent discharge. The seizures were partially controlled by phenobarbital. Craniocerebral ultrasound showed cerebral edema. Brain MRI showed diffuse signal abnormalities in bilateral cerebral hemispheres, basal ganglia, and thalamus (Figure 1Ab), and hence, a neurometabolic disorder was suspected. Fundus examination showed ischemic changes in the optic nerve in both eyes. Plasma amino acid and urinary organic acid profiles did not reveal any obvious abnormality. The following treatment measures were adopted for the patients: (1) Anti-infection treatment of ceftazidime, and the fluid volume was limited to 80–100 mL/kg/d; (2) Mannitol and furosemide were used to reduce intracranial pressure and brain edema; (3) Phenobarbital was used to control seizures in the early stages, following which, levetiracetam was applied. (4) Either oxygen or passive inhalation of oxygen was supplied; (5) L-carnitine and sodium bicarbonate infusion were given to correct acidosis; (6) Fasting was initiated, and then the low-protein milk powder was fed through the gastrointestinal tract. After 18 days of treatment, despite the difficulty in feeding (a tiny spoon feeding was necessary) and abnormal muscle tension, the infant showed the following symptoms: normal body temperature, steady breathing, flat bregma, seizure reduction, correction of acidosis, and decreased blood ammonia and lactate. Subsequently, it was instructed to continue feeding the patient with low-protein milk powder with oral administration of levetiracetam and levocanidin.

**TABLE 1:** Clinical features of the present cases and the reported in the literature.

Case	Sex	Onset age	Feeding problem	Seizures	Microcephaly	Lens subluxation	Extrapyramidal sign	Developmental delay	Dystonia	Outcome	Variants	References
1	M	16 days	+	+	+	−	+	+	+	9 months	c.475G > T(p.Glu159*) and c.1201A > G(p.Lys401Glu)	Present case
2	m	36 hours	+	+	+	+	+	+	+	32 months	c.571delC(p.Gln191Serfs*13), hom	Rupar et al., 1996
3	M	53 hours	+	+	NR	NR	NR	+	+	50 days	c.794C > A(p.Ala265Asp) and c.1280C > A(p.Ser427Tyr)	Edwards et al., 1999
4	F	24 hours	+	+	+	NR	NR	NR	+	10 weeks	c.571_574del CAGC (p.Gln191Glyfs*12), hom	Johnson et al., 2002b
5	M	21 days	+	+	NR	+	+	+	+	N/S	c.650G > A(p.Arg217Gln) and c.1200C > G(p.Tyr400*)	Lee et al., 2002
6	M	2 days	NR	+	+	−	NR	+	+	N/S	c.520delG (p.Asp174Thrfs*13), hom	Seidahmed et al., 2005
7	F	2 days	NR	+	+	+	NR	NR	+	14 months	c.1234_1235delGT (p.Val412Argfs*3), hom	Salih et al., 2013
8	M	3 days	+	+	+	NR	NR	+	+	Alive		
9	F	3 days	+	+	NR	NR	NR	NR	+	N/S	c.1200C > G (p.Tyr400*) + UPD (12) pat	Cho et al., 2013
10	M	1 day	NR	+	+	NR	NR	+	+	Alive	c.1136A > G (p.Lys379Arg), hom	Holder et al., 2014
11	F	14 hours	NR	+	NR	NR	NR	NR	NR	N/S	c.1200C > G (p.Tyr400*) and c.1355G > A (p.Gly452Asp)	Chen et al., 2014
12	F	1 days	+	+	+	−	+	+	+	N/S	c.1200C > G (p.Tyr400*), hom	Lee et al., 2017
13	M	3 weeks	+	+	+	NR	NR	NR	+	N/S	c.352C > T(p.His118Tyr) and c.649C > G (p.Arg217Gly)	Brumaru et al., 2017
14	M	3 days	+	+	+	NR	+	+	+	Alive	c.1084G > A(p.Gly362Ser), hom	Bender et al., 2019
15	M	15 days	+	+	+	−	+	+	+	N/S	c.884G > A(p.Gly295Glu), hom	Zaki et al., 2016
16	F	40 days	+	+	+	−	+	+	+	N/S		
17	F	24 hours	+	+	+	+	+	+	+	N/S	c.1313_1316delTAGA (p.Val438Aspfs*5), hom	Tan et al., 2005
18	F	2 days	NR	+	+	NR	+	+	+	Alive	c.1200C > G (p.Tyr400*) and c.1549_1574dup (p.Ile525Metfs*102)	Du et al., 2021
19	F	1 week	NR	+	NR	NR	+	+	+	9 years	c.1521_1524del TTGT(p.Cys508Argfs*109), hom	Mhanni et al., 2020
20	M	5 days	NR	+	NR	NR	+	+	+	15 months		
21*	F	5 months	+	+	NR	+	+	+	+	N/S	c.650G > A(p.Arg217Gln), hom	Garrett et al., 1998
22*	F	1 months	NR	NR	NR	NR	NR	+	+	Alive	c.427C > A (p.His143Asn), hom	Del Rizzo et al., 2013
23*	F	12 months	NR	NR	NR	+	+	+	+	Alive	c.182T > C (p.Leu61Pro) hom	Rocha et al., 2014
24*	M	2 years	NR	−	−	NR	NR	+	+	Alive	c.1096C > T (p.Arg366Cys) and c.1376G > A(p.Arg459Gln)	Tian et al., 2019
25*	F	14 months	NR	+	−	NR	+	+	+	Alive		
26*	M	16 months	NR	−	−	NR	+	+	+	Alive		
27*	M	9 months	NR	+	NR	−	+	+	+	Alive	c.1382A > T(p.Asp461Val), hom	Sharawat et al., 2020
28*	M	24 months	NR	+	−	NR	+	+	+	Alive		
29*	F	5 months	NR	+	+	+	NR	+	+	N/S	c.650G > A(p.Arg217Gln), hom	Lam et al., 2002

*NR, not report; +, present; -, absence; ^∗^, late-onset patients or mild patients; N/S, not specified in case report.*

A follow-up examination at the age of 5 months, he was presented with slow increase of body weight and progressive microcephaly. His weight was 5,500 g, and the head circumference was 40 cm which is 2 SD below the mean. Sucking and swallowing were significantly improved, but he also presented intermittent convulsions and axial dystonia. A repeated MRI on the same day showed polycystic encephalomalacia and atrophy with bilateral subdural effusion (Figure 1Ac). Serum ammonia and lactic acid levels returned to normal. Moreover, based on the genetic test results, we detected the level of *S*-sulfocysteine in patient’s urine and the level of t-homocysteine in patient’s dry blood spots by liquid chromatography-mass spectrometry (Shimadzu, Tokyo, Japan) (Fu et al., 2013; Sass et al., 2004). We also detected the level of uric acid in patient’s serum by uric acid method (Maccura, Chengdu, China), and the level of homocysteine in patient’s serum by cycling method (Gcell, Beijing, China) (Roberts and Roberts, 2004). The data showed that low total homocysteine was found in blood spot, while homocysteine and uric acid levels were normal in the plasma. *S*-sulfocysteine was abnormally elevated in urine (Table 2). The patient was treated with low methionine, low protein diet and low cysteine, and rehabilitation (dysphagia and sports) training was given. At 9 months, the patient died of worsened condition due to renewed fever and convulsions.

**TABLE 2:** Biochemical finding of the case.

Biochemical finding	Results	Reference value
*S*-sulfocysteine (urine)	112.786 μmol/mmolCrn	[Frame1]0.1–10 μmol/mmolCrn
Total-homocysteine (blood spot)	0.87 μmol/L	5–20 μmol/L
Homocysteine (plasma)	3.24 μmol/L	0–15 μmol/L
Uric acid (plasma)	227 μmol/L	210–430 μmol/L

### Genetic Analysis

Genomic DNA was extracted from 3 mL of peripheral blood leucocytes using the QIAamp Blood Midi Kit (Qiagen, Valencia, CA, United States), according to the manufacturer’s instructions. Whole exomes were captured (MyGenostics Inc., Beijing, China) and sequenced on Illumina HiSeq 2000 sequencer. Alignment and variant calling were performed by applying an in-house bioinformatics pipeline (MyGenostics). The variants with a minor allele frequency of <0.05 in population databases, such as 1,000 genome, ESP6500, dbSNP, EXAC, and in-house database (MyGenostics), expected to affect protein coding/splicing or present in the Human Gene Mutation Database (HGMD), were included in the analysis. The identified mutation was verified among the remaining family members by Sanger sequencing. The pathogenicity of candidate variants was deduced according to the American College of Medical Genetics and Genomics (ACMG) guidelines. The effect of missense variation on the three-dimensional (3D) structure of SUOX protein was analyzed by Swiss-PDB viewer (PDB: 1MJ4).

## Results

### Genetic Analysis and Co-segregation in the Family

Two novel variants were identified in the patient by NGS and bioinformatics analysis: a missense variant c.1201A > G(p.Lys401Glu) and a nonsense variant c.475G > T(p.Glu159^∗^) on exon 6 of the *SUOX* gene (NM_000456.3). Subsequently, the co-segregation verification of these two variants was demonstrated by Sanger sequencing. The father carried c.475G > T (p.Glu159^∗^) variant and the mother carried c.1201A > G (p.Lys401Glu) variant (Figure 1B). So the patient is compound heterozygous. None of these variants were reported previously. Both variants were absent in gnomAD (Supplementary Table 1) (PM2-Supporting). c.475G > T(p.Glu159^∗^) variant leads to premature termination of protein translation, which might damage gene function (PVS1); c.1201A > G(p.Lys401Glu) variant was detected in the *trans* position of the c.475G > T variant (PM3); c.1201A > G is predicted to be deleterious using multiple algorithm for missense mutation annotation (SIFT, PolyPhen-2, and MutationTaster) (PP3). According to the ACMG guidelines, c.475G > T(p.Glu159^∗^) is defined as “likely pathogenic” (PVS1 + PM2-Supporting) and c.1201A > G(p.Lys401Glu) as “uncertain significance” (PM3 + PP3 + PM2-Supporting) (Richards et al., 2015).

### Structure-Function Correlations of *SUOX* Variants

Sulfite oxidase is a homodimeric protein in the intermembrane space of mitochondria. It plays a vital role in the metabolic pathway of sulfur amino acids that are involved in the last step reaction in the oxidative degradation of the sulfur-containing amino acids, cysteine and methionine (Macleod et al., 1961; Feng et al., 2007). The SO deficiency prevents the sulfites from being oxidized to sulfates. The natural enzyme is a homodimer with a molecular mass of approximately 110 kDa. Each monomer include three different domains: a smaller N-terminal cytochrome b5 heme-binding domain, a central domain harboring the molybdenum cofactor (Moco), and a larger C-terminal dimerization domain with crucial residues at the dimer interface (Figure 1C) (Kisker et al., 1997). The nonsense variant p.Glu159^∗^ is harbored on the C-terminus of the cytochrome b5 heme-binding domain and near the beginning of the molybdopterin-binding domain of the SO, which might produce a truncated protein containing 159 amino acids, lacking a crucial molybdopterin-binding domain. The missense variant p.Lys401Glu is present in the last residue of the molybdopterin-binding domain, leading to the glutamic acid instead of lysine acid at position 401 in the SO protein. Reportedly, other missense variants (R160Q) in this domain can reduce enzyme activity (Garrett et al., 1998). Moreover, lysine 401 is conserved across evolution of SO (Figure 1D). SWISS-MODEL^1^ simulates the prominent amino acid and conformational changes in the influenced polypeptide (Figure 1E). Consequently, the length of the side chain was altered after the substitution of lysine by glutamic acid.

## Discussion

Moco is a core component of the sulfite oxidase maturation process. On the other hand, the synthesis of Moco requires several steps, the related enzymes are encoded by the genes *MOCS1*, *MOCS2*, *MOCS3*, and *GEPH*. Hence, the defect of Moco synthesis results in combined deficiencies of the enzymes SO, xanthine dehydrogenase, and aldehyde oxidase (Atwal and Scaglia, 2016). The two forms of SO deficiencies are regarded as Moco deficiency (MoCD) and ISOD, respectively. Nonetheless, these deficiencies are difficult to distinguish based on clinical manifestations. Biochemically, the affected individuals with ISOD and MoCD show the accumulation of sulfite, thiosulfate, and *S*-sulfocysteine in the tissues and body (Zaki et al., 2016). However, individuals with MoCD also display elevated urinary xanthine and hypoxanthine levels (Schwarz, 2005). In addition, urinary urothione, a breakdown product of the molybdenum cofactor, is absent in MoCD but present in ISOD (Sass et al., 2010). Therefore, genetic analysis is vital for the definite diagnosis of ISOD.

Most of the ISOD patients see a doctor in the neonatal period and the clinical manifestation is usually severe, including a progressive course with spasticity, intellectual deficit, microcephaly, and possible development of lens dislocation. In addition, ISOD is an incurable disease without an effective long-term therapy. Also, late-onset and mild forms of the illness have been described (Barbot et al., 1995; Touati et al., 2000; Del Rizzo et al., 2013; Rocha et al., 2014; Tian et al., 2019). The neuropathological characteristic of ISOD is significant but non-specific. The neuroimaging by computed tomography (CT) or MRI showed progressive neuropathological results, including cerebellar and cerebral atrophy, white matter changes, ventriculomegaly, and cystic leukomalacia (Claerhout et al., 2018). The clinical phenotype of our patient with ISOD was similar to that reported in the literature except for the absence of lens dislocation. Moreover, the results of the brain MRI showed progressive development; the MRI at the 5 months of age showed gradual polycystic encephalomalacia and atrophy with bilateral subdural effusion compared to that in the newborn. The natural history of ectopia lentis is difficult to describe because not all patients present lens subluxation in the first year of life (Lueder and Steiner, 1995). Our patient did not display ectopic lens but only ischemic changes in the optic nerve in both eyes, and the phenotype may or not appear with the age, thereby necessitating a regular follow-up. Biochemically, the patient presented low total homocysteine in the blood spot, while homocysteine and uric acid in plasma were normal. *S*-sulfocysteine presented an abnormally elevated level in urine. These clinical manifestations and laboratory results were in accordance with the diagnosis of ISOD.

Sulfite oxidase is a molybdo hemoprotein with a homodimer structure. Each monomer of SO contains three identical domains. Presently, the potential functionality of SO is not clear, but the dimerization of SO is crucial for a functional enzyme. Thus, mutations around the dimerization interface of SO result in the inactivation of the enzyme (Karakas and Kisker, 2005). In the central molybdenum domain, the pterin-based Moco forms the catalytic site of SO. Moreover, Moco is a vital constitute of the SO maturation process and a primary factor for heme integration and dimerization, further necessitating mitochondrial localization of SO (Atwal and Scaglia, 2016). The patient carried the heterozygous variant p.Lys401Glu, which is localized in the last residue of the molybdenum domain and adjacent to the dimer interface. Hence, we speculated that p.Lys401Glu affects the interaction between molybdenum and dimerization domains, which might disturb the structural stability of the protein. Thus, it is speculated that the positive charge lysine is replaced by the negative charge of glutamic acid, which might affect the binding of the enzyme active site. In addition, the lysine guanidino group might attract the divalent sulfite anion. The second novel variant p.Glu159^∗^ in the first domain of SO introduced stop codons and led to the premature termination of protein translation. Therefore, this variant led to a severe form of SO deficiency in our patient. Herein, we conducted genetic analysis on the family and identified that the variants, c.475G > T(p.Glu159^∗^) and c.1201A > G(p.Lys401Glu) derived from the father and mother, respectively. Genetic counseling is indispensable for the family which has a ISOD proband because the situation is often lethal in the neonatal period. Although the patient beyond the neonatal period, severe sequelae are unavoidable. In view of this situation, amniocentesis should be carried out between 15 and 23 weeks of the subsequent pregnancy in this couple for prenatal diagnosis (Özcan et al., 2017). The analysis of *SUOX* exon 6 is recommended to deduce whether the fetus carries any of the pathogenic variants from his parents.

The correlation between genotype and phenotype of ISOD has not yet been well elucidated. Reportedly, the clinical manifestations of patients with *SUOX* missense mutations were milder than those with null mutations (Claerhout et al., 2018), because these missense mutations of the *SUOX* gene only resulted in reduced enzyme synthesis, while null mutations abolished SUOX biosynthesis (Rocha et al., 2014). Herein, we reviewed 29 ISOD patients who reported genotypic and phenotypic features with integrity (Table 1); 20/29 patients were early-onset and 9/29 were late-onset or mild presentation. Interestingly, all the late-onset patients carried the missense variants that were distributed in the three structural domains of the SUOX protein. Conversely, among the 40 alleles carried by early-onset patients, nonsense variants accounted for 26/40 (65%) and missense variants accounted for 14/40 (35%) (Figure 1C). The age of onset ranged from 14h to 40 days in patients with early-onset or severe phenotypes, while it ranged from 1 month to 2 years in patients with late-onset or mild phenotypes and even in patients who spontaneously recovered without treatment. Therefore, the age at onset of ISOD patients may be related to the type of genetic variation. This conclusion provides a reasonable explanation for the clinical severity of our case.

To date, the treatment for neonatal ISOD is not promising. Typically, symptomatic treatment is primarily used to control seizures but with little success. However, dietary restriction intake of methionine, cysteine, and taurine has been found to be effective for mild patients with ISOD (Barbot et al., 1995; Touati et al., 2000; Del Rizzo et al., 2013; Rocha et al., 2014; Tian et al., 2019). In some circumstances, spontaneously recovery of late-onset mild ISOD has been reported (Tian et al., 2019). Belaidi et al. (2015) reported that the oxygen reactivity of mammalian SO provides a novel therapeutic route for the treatment of ISOD and MoCD. According to a recent study, oxidative stress and mitochondrial dysfunction underlie the pathophysiology of the brain damage of ISOD, providing novel viewpoints for the potential therapeutic strategies for this condition (Wyse et al., 2019). Thus, we tried low sulfur amino acid diet and oral levetiracetam, which improved the feeding difficulties; however, epilepsy did not improve significantly.

In conclusion, ISOD is a rare neurometabolic disorder that is difficult to diagnose by clinical symptoms alone. The two novel potentially pathogenic variants in *SUOX* were found in a Chinese mainland newborn patient with ISOD, and the clinical features were described comprehensively. Thus, the patients with suspected ISOD maybe more effectively diagnosed by genetic analysis, which would further improve the mutation spectrum of *SUOX*. In addition, genetic counseling is crucial because severe neurodegeneration develops, especially in the early neonatal period that prevents birth defects.

## Data Availability Statement

The original contributions presented in the study are included in the article/Supplementary Material, further inquiries can be directed to the corresponding author/s.

## Ethics Statement

The studies involving human participants were reviewed and approved by The Ethical Committee of the Xi’an Children’s Hospital. Written informed consent to participate in this study was provided by the participants’ legal guardian/next of kin.

## Author Contributions

JZ: conceptualization, writing manuscript, and editing. YA: data curation and sample sequencing. HJ: data curation, software, and methodology. HW: funding acquisition. FC: writing manuscript, editing, and manuscript review. YY: funding acquisition, project administration, and manuscript review. All authors contributed to the article and approved the submitted version.

## Conflict of Interest

The authors declare that the research was conducted in the absence of any commercial or financial relationships that could be construed as a potential conflict of interest.

## Footnotes

1 https://swissmodel.expasy.org
