# Supplementary material for: Novel Compound Heterozygous Pathogenic Variants in SUOX Cause Isolated Sulfite Oxidase Deficiency in a Chinese Han Family
Source: Front Genet. 2021 May 7;12:607085. doi: 10.3389/fgene.2021.607085 (PMC8139553; doi:10.3389/fgene.2021.607085)
Supplement: Supplementary file 1 [file Table_1.DOCX]

Table S1: All variants in the *SUOX* gene(NM_000456.3) was published in gnomAD database

| Nucleotide changes | Amino acid change | Domain | Allele Frequency | Heterozygote  Count | Homozygote Count | Reference |
| --- | --- | --- | --- | --- | --- | --- |
| **c.475G>T** | **p.Glu159*** | **Cyt-b5** | **0** | **0** | **0** | **Present case** |
| **c.1201A>G** | **p.Lys401Glu** | **MoCo** | **0** | **0** | **0** | **Present case** |
| c.182T>C | p.Leu61Pro | Transit peptide | 0 | 0 | 0 | Rocha,et al,2014 |
| c.287dupC | p.(Glu97*) | Cyt-b5 | 0 | 0 | 0 | Johnson,et al,2002 |
| c.352C>T | p.His118Tyr | Cyt-b5 | 0.000007976 | 2 | 0 | Brumaru,et al,2017 |
| c.427C>A | p.His143Asn | Cyt-b5 | 0 | 0 | 0 | Del Rizzo ,et al,2013; |
| c.520delG | p.(Asp174Thrfs*13) | Hinge | 0 | 0 | 0 | Seidahmed,et al,2005 |
| c.571_574del  CAGC | p.(Gln191Glyfs*12) | MoCo domain | 0 | 0 | 0 | Johnson,et al,2002 |
| c.571delC | p.(Gln191Serfs*13) | MoCo domain | 0.00003186 | 1 | 0 | Rupar,et al,1996 |
| c.649C>G | p.Arg217Gly | Molybdopterin-binding | 0 | 0 | 0 | Brumaru,et al,2017 |
| c.650G>A | p.Arg217Gln | Molybdopterin-binding | 0.00003181 | 8 | 0 | Kisker,et al,1997;  Lee,et al.2002;  Garrett,rt al,1998 |
| c.734_737del  TTTC | p.Leu245Profs*27 | MoCo domain | 0 | 0 | 0 | Johnson,et al,2002 |
| c.772A>C | p.IIe258Leu | MoCo domain | 0 | 0 | 0 | Johnson,et al,2002 |
| c.794C>A | p.Ala265Asp | MoCo domain | 0 | 0 | 0 | Kisker,et al,1997;  Edwards,et al,1999 |
| c.803G>A | p.Arg268Gln | MoCo domain | 0.000003977 | 1 | 0 | Johnson,et al,2002 |
| c.884G>A | p.Gly295Glu | MoCo domain | 0 | 0 | 0 | Zaki,et al,2016 |
| c.1084G>A | p.Gly362Ser | MoCo domain | 0.00003182 | 9 | 0 | Johnson,et al,2002 |
| c.1096C>T | p.Arg366Cys | MoCo domain | 0.00001193 | 3 | 0 | Tian,et al,2019 |
| c.1097G>A | p.Arg366His | MoCo domain | 0.00001193 | 3 | 0 | Johnson,et al,2002 |
| c.1126C>T | p.Arg376Cys | MoCo domain | 0.000007962 | 2 | 0 | Johnson,et al,2002 |
| c.1136A>G | p.Lys379Arg | Molybdopterin-binding | 0.000003978 | 1 | 0 | Johnson,et al,2002 |
| c.1187A>G | p.Gln396Arg | MoCo domain | 0 | 0 | 0 | Johnson,et al,2002 |
| c.1200C>G | p.Tyr400* | MoCo domain | 0.00006011 | 17 | 0 | Johnson,et al,2002 |
| c.1234_1235del  GT | p.Val412Argfs*3 | Homodimerization | 0 | 0 | 0 | Salih,et al,2013 |
| c.1261C>T | p.Gln421* | Homodimerization | 0 | 0 | 0 | Johnson,et al,2002 |
| c.1280C>A | p.Ser427Tyr | Homodimerization | 0 | 0 | 0 | Edwards,et al,1999 |
| c.1313_1316del  TAGA | p.Val438Aspfs*5 | Homodimerization | 0.00001414 | 4 | 0 | Tan,et al,2005 |
| c.1348T>C | p.Trp450Arg | Homodimerization | 0 | 0 | 0 | Johnson,et al,2002 |
| c.1355G>A | p.Gly452Asp | Homodimerization | 0.000007956 | 2 | 0 | Chen,et al,2014 |
| c.1376G>A | p.Arg459Gln | Homodimerization | 0.00001591 | 4 | 0 | Tian,et al,2019 |
| c.1521_1524del  TTGT | p.Cys508Argfs*109 | Homodimerization | 0 | 0 | 0 | Johnson,et al,2002  Mhanni AA,2020 |
| c.1549_1574dup | p.IIe525Metfs*  102 | Homodimerization | 0 | 0 | 0 | Du,et al,2020 |
| c.1589G>A | p.Gly530Asp | Homodimerization | 0.000003979 | 1 | 0 | Kisker,et al,1997 |
